# Cow's milk proteins allergy: the latest on therapy

**DOI:** 10.1186/1824-7288-41-S2-A29

**Published:** 2015-09-30

**Authors:** Marzia Duse, Lucia Leonardi

**Affiliations:** 1 Pediatric Department, “Sapienza” University, Rome, 00161, Italy

## 

The progressive increase in incidence in cow's milk proteins allergy (CMPA) in the past decades required primary prevention strategies for children at high-risk. Evidence of the role of gut microbiota in promoting the maturation of the immune system during early life encouraged supplementing the diet with probiotics in order to facilitate tolerance and delay or prevent sensitization. The efficacy of this strategy has not been consistently proven [1]. Breastfeeding is the most common therapeutic approach to CMPA in infants, although recent data showed that human milk has no effect on the development of allergy [2]. Use of special formulas is recommended in infants who are allergic or at high risk for CMPA. Extensively hydrolysed formulas (eHF) are the first therapeutic option. Amino acid-based formulas (AAFs) are recommended in infants who fail to respond to eHF, or have poor growth and IgE-mediated gastrointestinal disorders, or severe atopic eczema [3]. Hydrolysed formulas that do not originate from CMP are tolerated in 90% of children with CMPA. Use of soy milk is contraindicated in the first six months of life because of allergenic proteins and the presence of phytates and phytoestrogens [4]. The primary therapy for CMPA remains a strict avoidance of CMP, which promotes natural acquisition of tolerance in 80% of cases within the first 3 years of life. When the reintroduction of food causes severe clinical manifestations, diet restriction cannot be considered a solution, but instead exposes the infant to the constant danger of accidental exposure. Oral Immunotherapy (OIT) in food allergy requires the oral administration of increasing amounts of food up to a target dose, with the aim of reaching tolerance acquisition, which is considered complete when it is fully dose-independent[5]. OIT guidelines are not yet available. In severe, IgE-mediated clinical reactions to CMP, the combined therapy with Omalizumab, before and during “rush” OIT, reduced the number of severe adverse reactions and the duration of therapy while enhancing the possibilities of tolerance acquisition [6]. The use of Interferon-gamma (IFN-γ) administered subcutaneously, as adjuvant in oral immunotheraphy (OIT + IFN- γ), is a recent treatment. Unlike Omalizumab, the efficacy of IFN-γ has been demonstrated in non-IgE-mediated food allergy. IFN-γ seems to play a key role in the induction of tolerance but not in its maintenance. In studies on IgE-mediated CMPA, duration of treatment in patients with combined therapy (OIT + IFN-γ) is 2-3 months compared to 6-12 months for patients treated exclusively with OIT [7].
